# The Result of Removal of the Appendages

**Published:** 1899-04-29

**Authors:** 


### THE RESULT OF REMOVAL OF THE APPENDAGES.

In the choice of an operation for the relief of certain diseases of the female generative organs an argument founded on the retention or otherwise of sexual capacity is not infrequently brought into play; and this argument requires the most careful consideration, especially in view of the fact that it is one in regard to which accurate information is most difficult to obtain, owing to the reticence which most people prefer to maintain in reference to the whole subject. The mass of the profession must then of necessity trust in this matter to the opinion of experts, and unfortunately these do not all tell the same tale.

Writing on this subject in the New York *Medical Record*, Mr. Lawson Tait maintains that the position of those who think that in treating tubal disease it is an advantage so to arrange their operations as not to remove the tubes and ovaries, and who found this idea on the presumption that by so limiting their operations they will preserve the sexual instinct, is based on an entire misconception of the functions of these organs. These, he says, have nothing to do with the matter in question, and he adds: "Removal of the appendages does neither increase nor decrease sterility, and it often removes completely and permanently grave interference with marital life." As to the sterility it is easy to agree that, with our redundant population, it is almost a pity to encourage the production of children by those whose organs are in an abnormal condition. The question whether the instinct is preserved or abolished by removal of the appendages should, however, be decided by facts, and we can but take the word of those who have watched such cases. Mr. Lawson Tait says, "We are told once more, though the contrary has been proved over and over again, that in a considerable majority of cases there is a diminution or total abolition of the sexual instincts. This is not true; in fact, it is absolutely untrue. It is a subject on which, of course, the publication of facts is extremely difficult, either one way or the other. But my own facts establish the conclusion that the cases of abolition are extremely rare, not more than five per cent., but they get greatly talked about. . . . On the other hand, the instances of restitution of marital relations which had been entirely destroyed by disease and restored by the operation required are at least 60 per cent. of all the cases." This is a statement as to fact, and must be accepted as such. At the same time, we are bound to say that the cases which he gives as instances are not, to our mind, altogether satisfactory, being clearly abnormal, verging, indeed, on erotomania. The matter is an important one in this sense, that we ought to be prepared when the question is raised, and for this reason we have quoted Mr. Lawson Tait, although, of course, the existence of another and quite different view must be remembered. Whether the question should be raised at all, however, is quite another thing, and we may perhaps be allowed to express the opinion that such considerations should hardly be permitted to weigh seriously when the question under discussion is what operation to do upon a suffering woman. The great point to be considered should be, What will most safely remove her disease? and we have but little patience with the man who, in deciding what operation should be performed upon his wife, allows her hope of life to be in any way bartered against the chance of her being restored to the condition of a sexually perfect animal.

